# Significant Differences in Microbial Soil Properties, Stoichiometry and Tree Growth Occurred within 15 Years after Afforestation on Different Parent Material

**DOI:** 10.3390/life14091139

**Published:** 2024-09-09

**Authors:** Emre Babur

**Affiliations:** Faculty of Forestry, Kahramanmaras Sutcu Imam University, 46050 Kahramanmaras, Turkey; emrebabur@ksu.edu.tr

**Keywords:** parent material, afforestation, Scots pine, microbial biomass C, microbial respiration, plant growth

## Abstract

The mineralogical composition of the parent material, together with plant species and soil microorganisms, constitutes the foundational components of an ecosystem’s energy cycle. Afforestation in arid-semi arid regions plays a crucial role in preventing erosion and enhancing soil quality, offering significant economic and ecological benefits. This study evaluated the effects of afforestation and different parent materials on the physicochemical and microbiological properties of soils, including microbial basal respiration (MR), as well as how these changes in soil properties after 15 years influence plant growth. For this purpose, various soil physicochemical parameters, MR, soil microbial biomass carbon (C_mic_), stoichiometry (microbial quotient = C_mic_/C_org_ = *q*Mic and metabolic quotient = MR/C_mic_ = *q*CO_2_), and tree growth metrics such as height and diameter were measured. The results indicated that when the physicochemical and microbiological properties of soils from different bedrock types, along with the average values of tree growth parameters, were analyzed, afforestation areas with limestone bedrock performed better than those with andesite bedrock. Notably, sensitive microbial properties, such as C_mic_, MR, and *q*Mic, were positively influenced by afforestation. The highest values of C_mic_ (323 μg C g^−1^) and MR (1.3 CO_2_–C g^−1^ h^−1^) were recorded in soils derived from limestone. In contrast, the highest *q*CO_2_ was observed in the control plots of soils with andesite parent material (7.14). Considering all the measured soil properties, the samples can be ranked in the following order: limestone sample (LS) > andesite sample (AS) > limestone control (LC) > andesite control (AC). Similarly, considering measured plant growth parameters were ranked as LS > AS. As a result, the higher plant growth capacity and carbon retention of limestone soil indicate that it has high microbial biomass and microbial activity. This study emphasizes the importance of selecting suitable parent material and understanding soil properties to optimize future afforestation efforts on bare lands.

## 1. Introduction

Soil is formed under the influence of bedrock, climate, vegetation, and topographic factors. However, the dominance of one factor over the others can lead to variations in soil properties and land productivity [1]. Semi-arid and arid ecosystems, for example, are primarily influenced by parent material and the continental climate, with summer drought being a critical stress factor [2,3]. The physical properties, mineral composition, and texture of the parent material significantly affect the soil formation rate and the characteristics of the resulting soil [4]. The hardness of rocks is also crucial; soils formed on hard rocks have lower formation rates than the rate of soil loss through erosion, leading to poor habitat characteristics. Birkeland [5] ranked bedrock stability against soil formation factors as follows: quartzite, chert > granite, basalt > sandstone, siltstone > dolomite, limestone.

The parent material contributes to the nutrient cycle by releasing nutrients through weathering and maintaining a balance between nutrient loss and storage [1,4]. After climate, plant and microorganism activities play the most important roles in soil formation. Trees, in particular, influence parent material through biomechanical, chemical, and biological effects [1,3]. Traditionally, studies have focused on the biochemical, ecological, and edaphic influences on soils, but the significance of biomechanical effects is increasingly recognized [6,7,8]. Most research on the biomechanical effects of vegetation has centered on tree root growth, but trees also impact the physical displacement of regolith materials and the formation of root debris and exudates [9]. Plant roots mechanically break down rocks during growth, accelerating soil formation processes. Additionally, root exudates and mechanical root activities help reduce erosion, stabilizing soil ecosystems [10]. Organic acids and exudates produced by plants and microorganisms enhance the chemical weathering of minerals and the release of nutrients [11].

Over the last 20 years, the Turkish Ministry of Agriculture and Forestry has conducted afforestation activities to increase forest cover to 30% of Turkey’s surface area. Scots pine and black locust are commonly used for erosion control due to their high tolerance to drought and poor soil conditions [12]. Planting native tree species has accelerated natural succession [13] and carbon sequestration [14]. Plants, known as the organisms with the largest biomass in terrestrial ecosystems, contribute greatly to the formation and development of soils through litter residues, leaves, branches, roots, and exudates of their aboveground biomass [15,16]. In addition, vegetation affects the organic carbon (OC) concentration added to soils and the diversity, quality, and quantity of soil microorganisms [17]. For example, the biomass of microbial organisms in soils converted from forest to agriculture decreases significantly [18,19]. Similarly, agricultural land contains less soil organic C (SOC) and C_mic_ compared to native forests [20]. Vegetation type strongly influences soil-forming processes, contributing significantly to organic matter and microbial community development and activity [21,22]. Microbial stoichiometric indices such as microbial basal respiration (MR), microbial quotient (*q*Mic), metabolic quotient (*q*CO_2_), and C mineralization are related to the SOC cycle in the environment. With the help of these stoichiometric indices, the effects of changes in land use or ecosystem on soil quality, microbial populations, and microbial activity rates can be estimated [23,24].

While many studies suggest that microbial biomass is controlled by soil physicochemical properties [25,26], others indicate that the aboveground vegetation type has a stronger influence on microbial biomass and activity [27,28]. Kara et al. [10] found positive effects of ecosystem changes on microbial biomass and activity in afforested areas in both the short and long term. Studies on afforested ecosystems indicate that vegetation accelerates soil formation processes, promoting organic matter accumulation and the development of microbial communities [21,29,30]. Trees provide nutrients to the soil through dead plant residues and root secretions, which microorganisms break down and mineralize, forming a usable nutrient source for plants [31]. Microbial organisms decompose this litter, returning essential organic nutrients to the soil. This process enriches the growing environment, enhancing the productivity of the soil. As a result, plant growth parameters, such as tree height (TH) and diameter at breast height (DBH), improve significantly. The upper soil horizon, a reservoir for soil microbial biomass, is crucial for decomposition and nutrient cycling [32]. This symbiotic relationship underpins the nutrient cycle, essential for ecosystem health.

Soil microorganisms are the most rapidly affected by environmental conditions, various forestry management practices, and sudden changes on or within the soil surface. Consequently, measuring microbial activities and properties following forestry management activities is essential for monitoring changes in soil fertility. Changes in microbial properties can provide early indications of potential ecosystem disruptions [31,33,34,35,36]. Assessing microbial biomass in soils is crucial in many soil monitoring studies and programs [34], but it should be considered alongside other habitat parameters for a comprehensive ecological assessment. Although recent studies focus on the effects of soil microbiological properties on soil fertility, there is still limited information on their relationship with tree growth. This research aimed to new perspectives on the effects of physicochemical, microbial properties, and stoichiometry of soils derived from different bedrocks on seedling growth parameters, highlighting the importance of evaluating soil quality parameters in forests.

## 2. Materials and Methods

### 2.1. Study Sites

The study was conducted in Gümüşhane Province, located in the Northeast Black Sea mountainous region of Turkey (40°19′–40°23′ N and 39°30′–39°38′ E). The elevation ranges from 1400 to 1600 m, with steep slopes (45–50%) covered by shallow soil (typically 50 cm or less) on a west-southwest aspect. The region has a “Warm, dry-summer continental climate” (Dsb) according to the Köppen-Geiger climate classification (https://koeppen-geiger.vu-wien.ac.at/; accessed on 1 July 2024). Weather data, including long-term averages (1990–2018) for monthly air temperature and precipitation in Gümüşhane Province, were obtained from the Turkish State Meteorological Service (www.mgm.gov.tr; accessed on 20 June 2024). The annual mean temperature is 9.4 °C, with a high of 39.5 °C in July and a low of −19.0 °C in February. The topsoil temperature drops below 0 °C and freezes during the winter. Annual precipitation is 461 mm, with about 54% occurring during the growing season between April and September. The main soil type in the area is Entisol, characterized by minimal horizon development. The rugged topography consists of andesitic and limestone bedrock. Table 1 provides a detailed characterization of the study site.

### 2.2. Experimental Design, Soil Sampling, and Pretreatment

This study was conducted in Scots pine (*Pinus sylvestris* L.) afforestation areas in June 2019. These areas were afforested by the Turkish General Directorate of Afforestation in 2005, initially prepared with an excavator, and the seedlings were supported with irrigation and fertilization for the first five years after planting. The control area was severely degraded grassland with less than 5% vegetation cover, exposed to erosion, and left unfertilized. A grid-based sampling design was used to select three 10 m × 10 m experimental areas from different bedrocks, representing a total of 30 hectares [37]. Tree growth parameters, such as tree height (TH) and diameter of breast height (DBH), were directly measured within each 100 m^2^ area using a Blume-Leiss and compass, respectively. TH and DBH were measured in 53 trees (in andesite) and 64 trees (in limestone) within the afforested study site. Soil samples were collected from Scots pine plantations and adjacent bare control plots during periods of optimal moisture and temperature for microbial activity. In particular, clearings adjacent to afforestation areas were selected as control areas to better identify the differences in bedrock and afforestation effects. The sampling locations were on afforested terraces within 10 m × 10 m plots. For each parent material, a total of 10 soil samples were taken, as two of the selected trial sites with different parent materials had three terrace rows, while one had four terrace rows. A total of 40 soil samples (4 different sites × 10 spots = 40 soil samples) were systematically selected from two different parent materials afforested (LS = limestone sample and AS = Andesite sample) and control areas (LC = limestone control and AC = Andesite control). The disturbed and microbial soil samples were collected separately from the topsoil layer at 0–20 cm depth. Before collecting disturbed mineral soil samples, the litter, root debris, and stones were removed and stored in plastic bags. In the field, microbial soil samples were first sieved using <2 mm sieve, then distilled water was added until 50–60% moisture content to ensure optimal conditions for microbial activity, which is crucial for accurate measurement of microbial properties and quickly transferred to the laboratory for storage in a 4 °C refrigerator before analysis [22]. Disturbed soil samples were dried by spreading them in drying cabinets to make them air-dried; after drying, the soils were ground and sieved (<2 mm) to determine the physicochemical parameters of the soil.

### 2.3. Physical and Chemical Properties of Soils

Soil moisture content was determined gravimetrically by oven-drying samples at 105 °C until a stable weight was achieved. Soil particle size (PS) was measured using the hydrometer method [38]. Soil pH and electrical conductivity (EC) were assessed using a 1:2.5 soil/water suspension for pH and a 1:5 soil/water suspension for EC, measured with appropriate meters. Soil organic carbon (SOC) was estimated using the potassium dichromate oxidation method [39], total nitrogen by the Kjeldahl method [40], and total lime (CaCO_3_) content was determined using the Scheibler calcimeter method [41].

### 2.4. Microbial Biomass C (C_mic_)

The chloroform-fumigation-extraction method was performed to determine soil microbial biomass carbon (C_mic_) [42]. For microbial analysis, 30 g of each soil sample was taken in two replicates and fumigated with ethanol-free chloroform in a heavy vacuum desiccator at 25 ° C for 24 h. Similarly, the second part of the microbial soil samples weighed 30 g and were not fumigated with chloroform in the desiccator. Both fumigated and non-fumigated soil samples were mixed in 0.5 M K_2_SO_4_ (1:4 *w*/*v*), followed by shaking the suspension for 30 min at 200 rpm in an oscillator. The extracts were filtered through the Whatman-42 filter paper and stored before titration. Total organic carbon in both fumigated and non-fumigated extracts was estimated as specified by the Walkley Black method [43,44]. C_mic_ was calculated from the difference in extractable organic C between fumigated and unfumigated soil samples using the formula: biomass C = 2.64 × EC, where EC refers to the difference in extractable organic C between the treatments; 2.64 is the proportionality factor for biomass C released by fumigation extraction [42,44]. The microbial quotient (C_mic_/C_org_ or *q*Mic) was calculated by expressing microbial C as a percentage of total soil organic C.

### 2.5. Microbial Respiration

Microbial respiration was determined using the absorption method to quantify carbon dioxide (CO_2_) [45]. Moist soil samples (50 g dry weight equivalent) were placed in 60-mL beakers and incubated in the dark at 25 °C in 500 mL airtight, sealed jars along with 10 mL 0.1 M NaOH. After 7-day incubation, the CO_2_ generated was measured by titration of the excess NaOH with 0.05 M HCl [46]. An empty jar without soil was used as the control. The difference in the volume of HCl consumed between the treatment and the control was used to calculate CO_2_ emission from soil microorganisms. The metabolic quotient *q*CO_2_ is an important parameter that can be used to estimate soil quality and stress response. It was calculated as the microbial respiration rate (μg CO_2_–C h^−1^) per mg of C_mic_ [45].

### 2.6. Statistical Analyses

The data were tested for normality and homogeneity of distribution using skewness, kurtosis, and Shapiro–Wilk tests. Parameters that deviated from normal distribution were log-transformed (log10) before analyzing variance. The skewness and kurtosis values were between −2 and +2, indicating that the variables were normally distributed [47]. In the study, while parent material (Andesite and Limestone) and location (control and afforested) factors were taken in the statistical model, it was investigated whether there was a statistical difference between the application and bedrock averages and the existence of an interaction. Therefore, parametric test techniques were applied: an independent T-test was used for comparisons of the means of DBH and TH characteristics of pine in different parent material, and the two-way ANOVA (two-way Analysis of Variance) test was applied to the penetration area and maximum penetration properties and the sand, clay and silt percentage properties that did not meet the prerequisites of the parametric tests, after applying the inverse angle (arcsin) transformation, followed by Duncan’s multiple comparison tests (α = 0.05) for comparisons independent variables. After confirming that the data met the assumptions for parametric tests, Pearson’s linear correlation analysis was also conducted to assess the relationships between soil properties and plant growth characteristics. Discriminant analysis (DA) was employed to analyze the differences between groups of multivariate data using one or more discriminant functions to maximally separate the identified groups. The statistical analyses were carried out using the R studio 20.0 (R Core Team) and Statistical Package for the Social Sciences 22.0 (SPSS Inc., Chicago, IL, USA) and figures were generated by using the XLSTAT Windows 2018 software package.

## 3. Results

Under similar ecological conditions, soils formed from different bedrocks exhibited significant differences in physical and chemical properties, including sand, silt, clay content, pH, EC, SOC, TN, and C/N ratio. These variations also extended to microbial activity indicators such as MR, C_mic_, qMic, and *q*CO_2_. Consequently, these differences in soil properties and microbial activity led to significant variations in the growth characteristics of the seedlings planted in the area (Table 2, Table 3 and Table 4).

### 3.1. Physicochemical Properties of the Soil

The results indicated significant differences in the analyzed properties of soils developed on different parent materials, with notable improvements in soils from post-afforestation sites. The two-way ANOVA test revealed that these soils’ physical, chemical, and microbiological properties were significantly different from each other and the control areas. These differences in soil properties influenced soil fertility, resulting in varying development rates among the seedlings planted in the various areas. The mean values of some physicochemical soil properties are presented in Table 2 and Table 3.

According to international soil texture classifications [48], the soils at the research sites displayed varying textures based on parent material. In the afforestation site, soils derived from limestone were classified as loamy clay, while those in the limestone control site were also clay loam. Soils from andesite were identified as sandy clay loam in the afforestation sites and sandy loam in the control area. The percentages of sand, silt, and clay differed significantly depending on the parent material. Soils from andesite had significantly higher sand content (75.25 ± 0.41 in AC and 72.58 ± 0.67 in AS) compared to those from limestone (Table 2). The difference between the average sand ratio of andesite (72.58 ± 0.674) and the average limestone sand ratio (53.50 ± 0.297) in afforested areas is statistically significant (*p* < 0.05). Similarly, the difference between the average andesite and limestone sand ratio in control areas is statistically significant (*p* < 0.05) (Table 3). Conversely, the average silt and clay contents were significantly higher in limestone-derived soils (Table 2).

The mean soil reaction (pH) values of the limestone soils statistically differed from the andesite soils (*p* < 0.001) (Table 3). The mean pH value in all soil samples, including controls, was found to be the highest in the LC site (Table 2). However, the lowest mean pH value was observed in the soils of the areas with AC location. Moreover, the mean pH’s (8.05 ± 0.031 and 8.17 ± 0.027) in the LS sites were higher than AS and AC sites, with 7% and 8%, respectively. The pH classification of limestone bedrock was moderately alkaline in afforestation and control sites and slightly alkaline in andesite bedrock afforestation (7.59) and control (7.56) sites. While the mean electrical conductivity (EC) values were found to be statistically different in different parent materials (*p* < 0.05), they were not different for locations (*p* > 0.05) (Table 3). On the other hand, EC values of LS soils were found to be 30% higher than AC soils and 42% higher than AS soils (Table 2).

As expected, SOC and TN contents differed significantly among parent materials and location levels (*p* < 0.001), but no statistically significant difference was found in the mean SOC and TN values of control plots (*p* > 0.05). The average SOC contents of the afforestation sites were 1.65% in andesite and 2.40% in limestone, while TN content was 0.10% in andesite and 0.19% in limestone. SOC and TN amounts in afforestation sites increased by 300% in limestone and 333% in andesite compared to control sites. These results indicate that SOC and TN levels were highest in LS (Table 2). The highest annual mean C/N ratio was observed in the AC site (22.74 ± 0.99), followed by the LC (18.76 ± 0.36), the AS, and finally, the lowest in the LS (12.61 ± 0.59). The mean C/N ratio of the AC site was 17%, 29%, and 44% higher than the LC, AS, and LS sites, respectively (Table 2). Additionally, the soil CaCO_3_ percentage was significantly higher in soils derived from limestone compared to andesite. After afforestation, this value decreased by 30% in limestone parent material and by 36% in andesite parent material compared to the control sites.

### 3.2. Soil Microbiological Properties and Stoichiometric Indices

The significant changes were determined in the C_mic_ values of the areas after afforestation. The highest average C_mic_ values in soil samples were found in the LS site (323.20 ± 20.50 μg C g^−1^), while the lowest was found in the AC site (35.09 ± 1.09 μg C g^−1^) (Table 2). In addition, statistically significant differences were determined between the C_mic_ amount of afforested and control areas in andesite locations (*p* < 0.001) (Table 3). Similarly, the highest average microbial respiration (MR) rate (1.30 ± 0.08 μg CO_2_–C g^−1^ h^−1^) from different soil samples was found in LS soil, followed by AS (0.62 ± 0.05 μg CO_2_–C g^−1^ h^−1^), LC (0.35 ± 0.01 μg CO_2_–C g^−1^ h^−1^) and AC (0.25 ± 0.008 μg CO_2_–C g^−1^ h^−1^), respectively (Table 2). C_mic_ and MR values of soils increased by approximately 530% with afforestation on both parent materials. The highest mean value of the C_mic_/C_org_ (*q*Mic) ratio was found in the LS site at 1.39 ± 0.07, while the lowest value was observed in AC sites at 0.59 ± 0.03 (Table 2). MR, C_mic_, and *q*Mic values were ranked from largest to smallest as LS > AS > LC > AC. The highest and lowest *q*CO2 values of soil samples were recorded in AC (7.14 ± 0.24) and AS (3.37 ± 0.27) sites, respectively (Table 2). The *q*CO_2_ values of control sites were found to be significantly higher than afforested sites. Additionally, significant statistical differences were found between the *q*CO2 amounts of afforested areas in andesite locations and control areas (*p* < 0.001) (Table 3). The *q*CO2 index order is AC > LC > LS > AS, unlike other microbial properties and indices.

### 3.3. Plant Growth Characteristics

**Figure 1:**
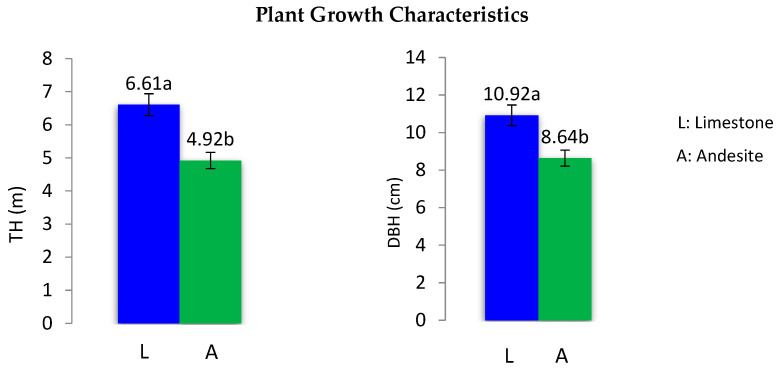
Changes in plant growth characteristics in different parent material sites. Different letters above the bars indicate significant differences at *p* < 0.05 among the land use types.

Independent T-test revealed that plant growth traits were significantly different according to parent materials. The average diameter at breast height (DBH) of the saplings on limestone bedrock was 10.92 cm, compared to 8.64 cm on andesite parent material. Similarly, the average tree height (TH) of saplings on limestone parent material was 6.61 m, while it was 5.54 m on andesite parent material (Table 4 and Figure 1). These results indicate that, following afforestation, the average height and diameter of saplings on limestone parent material were 20% and 25% higher, respectively, than those on andesite parent material, despite the presence of the same tree species and ecological conditions (Figure 1).

**Figure 2:**
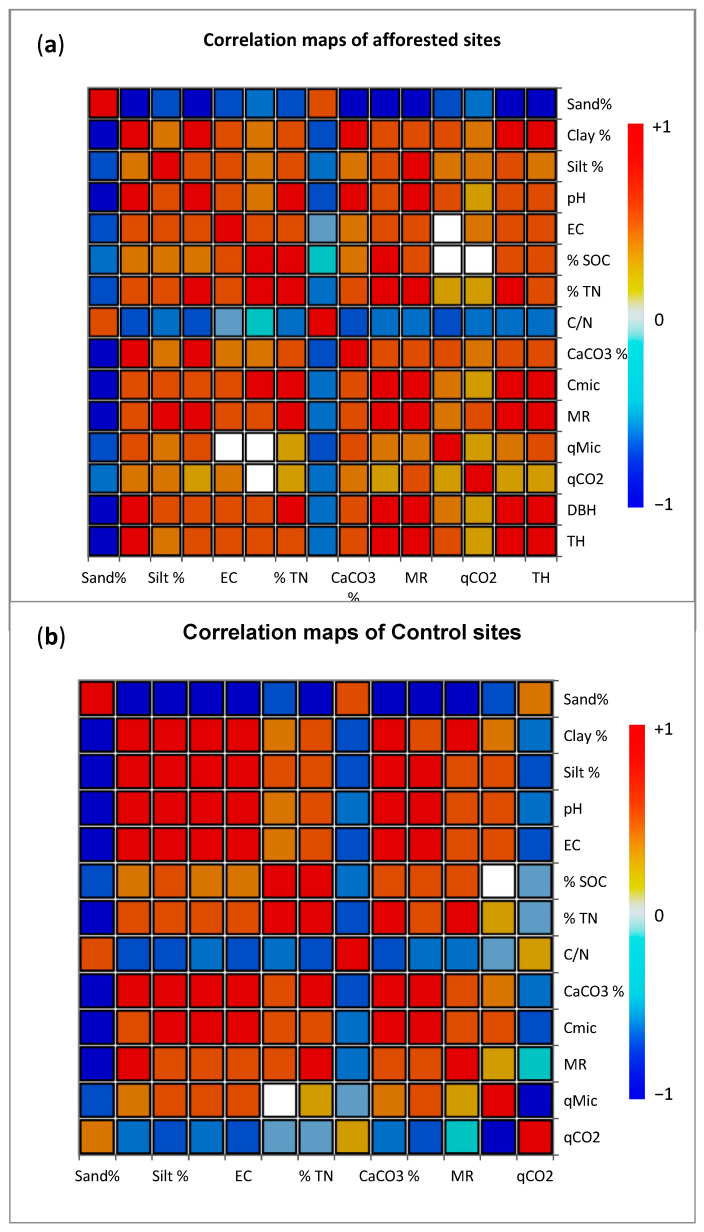
Correlation maps of soil samples of different parent material afforested (**a**) and control (**b**) sites according to correlation coefficient matrix (R-values) for physical, chemical, and microbiological characteristics of soils in different land uses.

The Pearson correlation coefficients between TH, DBH, C_mic_, MR, *q*Mic, *q*CO_2_, SOC, TN, pH, EC, CaCO_3_, sand, silt, and clay were calculated for both afforested and control areas (Figure 2). It was determined that there were positive correlations between TH and dust, clay, pH, EC, SOC, TN, CaCO_3_, C_mic_, and DBH (*p* ≤ 0.001). The highest positive correlations with TH were shown by clay, CaCO_3_, DBH, and Cmic, respectively. Again, the highest positive correlation values were found between DBH and CaCO_3_, C_mic_, MR, and Clay, from largest to smallest, respectively. Here, it was determined that microbial parameters had a positive effect on plant growth parameters, at least at the significance level of 0.05. In addition, all of the physicochemical properties of the soils, except sand and C/N ratio, showed positive correlations with plant growth parameters. Other interesting positive correlation values were found between C_mic_, SOC, and TN. On the other hand, the highest negative correlations were found between plant growth parameters and sand and C/N (Figure 2a).

In addition, when the relationships of soil properties in control areas belonging to different bedrocks were examined according to Pearson correlation, it was determined that sand, C/N, and *q*CO_2_ values had a significant negative relationship with clay, silt, pH, EC, CaCO_3_, C_mic_, MR, *q*Mic. Except for *q*CO_2_, the microbial indices of the soil showed the highest positive correlation with clay, dust, and CaCO_3_ among the physicochemical properties (*p* < 0.001) (Figure 2b).

**Figure 3:**
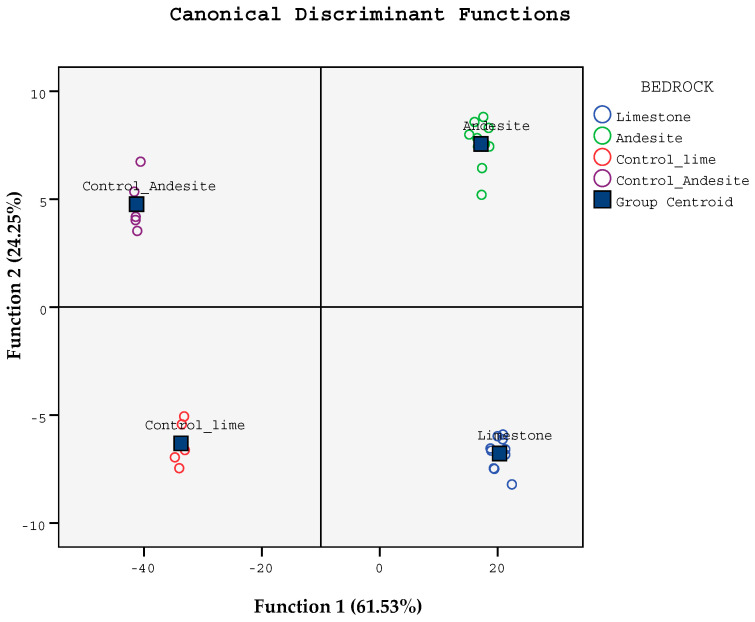
Scatter plot of the first two discriminant function values in soil samples.

Discriminant analysis (DA) was able to distinguish between soils formed from limestone and andesite bedrocks in terms of physicochemical, microbiological properties, and plant growth parameters (Figure 3). The soil variables contributing to the classification and the values of the three canonical functions are shown in Table 4. Evaluations were made by accepting significant values above 0.3 [49]. Eigenvalues for the first two axes in all soil samples were 1.027 and 0.841, respectively, and explained 61.53% and 24.25% of the total variance (Figure 3). Correlation coefficients show that DA axis 1 has the strongest correlation with *q*Mic, C/N, *q*CO_2_, TH, DBH, and clay, respectively. DA axis 2 is seen to be mainly related to SOC, TN, C_mic_, and MR, respectively, with the strongest correlation (Figure 3). Standardized canonical discriminant function coefficients of soil properties are given in Table 5.

## 4. Discussion

In this study, it was revealed that physicochemical and microbiological soil properties, as well as plant growth parameters, were significantly affected by the type of parent material. Limestone soils formed fine-grained soils with significantly lower sand and higher silt and clay. In other words, andesite produced coarse-grained soils with significantly higher sand and lower silt and clay percentages. Notably, the afforestation sites decreased sand and dust percentages compared to the control sites, while the clay percentages increased (Table 2). Some studies indicated that soils developed from limestone bedrock had lower sand and higher clay and silt contents than other bedrock types, such as granite acrostic [4] and andesite [1]. Similarly, ref. [3] determined that the grain sizes of soils from granite, granodiorite, and schist bedrocks were different.

The parent material difference also influenced the chemical properties of soils, such as pH, EC, SOC, TN, and CaCO_3_, in both control and afforestation sites. Previous studies have reported significant variations in soil pH values due to differences in parent material [1,3,4]. The EC values of soils from both parent materials at the study sites were below the critical salinity level of 0.20 dS m^−1^. SOC and TN content differed significantly depending on the parent material and were higher in afforestation sites compared to control sites. The increase in carbon and nitrogen sources in soils after afforestation affects the quality and quantity of organic material, including litter production [50], root exudates [51], and microbial community structure and activity [15]. Plant residues such as leaves, cones, and branches contribute to increased SOC and TN levels in the ecosystem [31,52,53]. Moreover, factors like higher clay content, tree presence [54,55,56], root exudates, and plant residues [15] promote soil formation. While the effect of bedrock on soil properties was observed in control sites, afforestation, particularly with Scots pine, appears to have significantly contributed to the increases in SOC and TN. The concentration of SOC and TN in the study area may be lower than in mature forests, as only fifteen years have passed since afforestation. It may take a longer period for litter to accumulate on the soil surface and integrate into the soil. Typically, SOC and TN concentrations are higher in the topsoil layer (0–20 cm) due to litter accumulation [4,31,32]. As expected, CaCO_3_ content was significantly higher in soils with limestone parent material, attributed to the inherent lime content of limestone. There was a notable decrease in CaCO_3_ content after afforestation, likely due to plant influence on soil formation. It has been reported that afforestation with different species can reduce the amount of lime in the soil, particularly in the topsoil [1]. Limestone soils are characterized by alkaline properties and texture variations between sand (sandstone) and clay (shale) [57].

The chemical properties of soils, such as pH, SOC, and TN, and microbial properties of C_mic_, N_mic_, MR, and other indices are important sensitive indicators affected by several factors such as seasonality, tree species, bedrock properties, and soil properties [16,58]. The available C_mic_ in soils directly indicates the microbial biomass pool in the soil. The study results illustrated that the parent material significantly affects the C_mic_ concentrations in soils. Mahia et al. [50] stated that schist was significantly different from granite parent material in C_mic_, while Babur [1] reported that soils developed from limestone parent material had higher C_mic_ than andesite. In addition, the afforestation of bare land contributes to the nutrient cycle of the ecosystem, ecosystem productivity, soil quality, and the increase in the amount of soluble and storable organic C in the soil with the input of organic matter from litter and dead roots [59,60]. Increased organic carbon in soil also affects the biological properties of soils [61]. Soil C_mic_ is generally dependent on soil organic matter as a substrate [24]. In addition, exudates brought to the soil by plant roots can increase C_mic_ accumulation, especially in topsoil [62]. In this article, it was confirmed that the increased organic matter input after afforestation also significantly increased the C_mic_ amount.

Microbial respiration (MR) is a key factor in monitoring decomposition processes in soils [34,46]. MR reflects the oxidation status of the soil by microbes and is considered one of the most important and sensitive indicators of the soil carbon cycle [63,64]. Babur et al. [32] found significant differences in microbial biomass carbon (C_mic_) and MR values among different tree species. Similarly, it was reported that optimum soil temperature and moisture content, which enhance microbial activities in different tree plantations, typically occur during rainy seasons [65].

The *q*Mic values of all soil samples were found to be within the range suggested by Anderson and Domsch [66]. *q*Mic can be used to quickly estimate the change in organic C [67] and to compare soil quality parameters in ecosystems with different organic matter [61]. The *q*Mic ratio is determined by using the relationship between microbial biomass carbon and soil organic carbon [68]. The *q*Mic ratio of andesite soils of the study areas was lower than that of limestone. This provided more suitable conditions for the growth of soil microorganisms in the limestone bedrock. Although the average *q*Mic values increased after afforestation, this value remained low for fertile lands and did not reach equilibrium 15 years after afforestation. The *q*Mic value is estimated by microbial activity, i.e., soil basal respiration and organic carbon cycling within an ecosystem [34,69]. Higher MR and C_mic_ amounts in limestone soils indicated that it had more microbial activity, proving faster decomposition.

The *q*CO_2_ is often used to describe the soil stress response of an ecosystem [69]. Environmental stresses, nutrient scarcity, and substrate resistance to decomposition can increase *q*CO_2_ [70]. On the other hand, the productivity of the area can be increased by using natural vegetation in stressed areas [71]. Although Anderson [69] reported that *q*CO_2_ values in neutral soils ranged from 0.5 to 2.0 μg CO_2_-C g^−1^C_mic_ h^−1^, in this study, the average *q*CO_2_ values in areas afforested with andesite and limestone bedrocks were found to be 3.37 to 4.06 μg CO_2_-C g^−1^C_mic_ h^−1^, respectively. This may cause microbial respiration to increase, decomposition to slow down, and energy to be consumed in it. Low *q*CO_2_ rate in soils provides positive information about efficient carbon use for that soil, low environmental stress levels, usable nutrients being free in the area, microbial activity, and soil quality [22].

The *q*CO_2_ and OC mineralization rates in soil constitute the basis of the carbon cycle in terrestrial ecosystems [10,72]. SOC content negatively correlated with the metabolic coefficient in both parent materials. Some studies have noted that OC and *q*CO_2_ in different forest soils are closely related [1,62]. As a result of the correlation analysis, it was determined that basal respiration was positively correlated with soil organic carbon in both parent materials. Similar results were found with Cheng and Xia [73].

Differences in parent material and soil properties showed significant differences in plant growth parameters such as TH and DBH. In particular, Scots pines on limestone bedrock were 20% and 25% larger in height and diameter than on andesite bedrock, respectively. There are many studies on the importance of the effects of habitat properties on plant yield and growth characteristics [74,75,76]. Microbiological, physical, and chemical soil properties can improve as the age of the afforested area. Afforestation and rehabilitation practices in forest management contribute to the health and productivity of soils and support the restoration of soil microbial activities [10,52].

Available C fractions are one of the most important environmental factors affecting soil microbial activity [22,59]. Analyses performed on soil samples, particularly microbial biomass, MR, and other indices, may be responsible for the separation of different parent materials and control areas with DA because the parent material is an important factor in soil formation, and tall plants also provide more living space and substrate for microbial communities in soils with their aboveground and belowground biomass and waste. Improvement of microorganisms’ living environments can also be understood by the increase in *q*Mic value and decrease in *q*CO_2_ value [77,78]. In fact, from our research, it was determined that there was an increase in *q*Mic amount and a decrease in *q*CO_2_ amount in soils on both bedrocks after afforestation. Both growth data (DBH and TH) were positively correlated with all soil properties except sand percentage and C/N. These results provide strong evidence that afforestation on different bedrocks results in different soil properties and microbial communities compared to bare lands.

## 5. Conclusions

In this study, it was determined that the physicochemical and microbiological properties of soils formed from different parent materials were different, and this significantly affected the growth parameters of the planted seedlings. It was determined that the fertility of soils in terrestrial ecosystems was significantly affected primarily by the type of parent material and then by the presence of vegetation on it. The fact that limestone parent rock can be easily decomposed compared to andesite and that it creates deep and fertile soil allowed Scots pine trees to grow better on limestone parent rocks. It was shown that afforestation contributed to the increase in organic C and N, especially in the upper soils in bare lands, and this contributed to the increase in microbial biomass and activity in the soils. Soils from limestone parent rocks afforested with Scots pine were energetically more efficient (had a lower *q*CO_2_) with more microbial biomass and a higher *q*Mic (C_mic_/C_org_) compared to andesite soils. This study emphasized how important parent material is in terrestrial ecosystems and that it directly affects soil microbial activity. In particular, afforestation provides substrate and C to the ecosystem via tree litter, thus increasing soil microbial communities and activities. Since the measured microbial parameters are sensitive to changes in the ecosystem, they may have the potential to be used as indicators of forest management on soil organic matter quality. In order to ensure data continuity in such areas and to verify the data of afforestation sites in arid and semi-arid areas, more comprehensive studies are required. In afforestation to be made on andesite bedrocks, more care should be taken, and maintenance, fertilization, and even irrigation during periods of water stress can contribute to the growth of seedlings and environmental fertility. In the afforestation of arid and semi-arid areas, should be paid to selecting local species and close origins. In addition, it would be more appropriate to make afforestation using mixed species with leaves and conifers to increase the ecological sensitivity limit. Thus, ecosystem diversity, nutrient cycle, and soil C input will be supported.

## Figures and Tables

**Table 1:** Study site characteristics.

Environmental Factors	Limestone		Andesite	
	Sample	Control	Sample	Control
Longitude	39°33′19″	39°33′46″	39°33′43″	39°33′23″
Latitude	40°21′08″	40°21′23″	40°21′20″	40°21′07″
Altitude mean (m)	1480	1520	1500	1450
Slope degree mean (%)	50	40	45	50
Afforestation date	2005	-	2005	-
Vegetation type	Scots Pine	Bare space	Scots Pine	Bare space
Stand canopy (%)	70–100	-	70–100	-
Soil texture	Loamy clay	Clay loam	Sandy clay loam	Sandy loam
pH class	Moderately Alkaline	Slightly Alkaline	Moderately Alkaline	Slightly Alkaline

**Table 2:** Mean ± standard deviation and Duncan multiple comparison results for the soil properties considered for each parent material and location.

Soil Properties	Locations	Parent Material		Parent Material Mean ± SE
		Andesite	Limestone	
Sand (%)	Afforested	72.6 ± 0.674 ^Ab^	53.5 ± 0.297 ^Bb^	63.04 ± 2.2
	Control	75.3 ± 0.408 ^Aa^	59.7 ± 0.286 ^Ba^	67.47 ± 1.8
	Location Mean ± SE	73.9 ± 0.491	56.6 ± 0.739	
Clay (%)	Afforested	16.8 ± 0.683 ^Ba^	31.1 ± 0.840 ^Aa^	23.96 ± 1.7
	Control	11.1 ± 0.467 ^Bb^	18.4 ± 0.429 ^Ab^	14.77 ± 0.89
	Location Mean ± SE	13.98 ± 0.766	24.75 ± 1.530	
Silt (%)	Afforested	10.6 ± 0.334 ^Bb^	15.40 ± 0.918 ^Aa^	13.00 ± 0.73
	Control	13.7 ± 0.255 ^Bb^	21.90 ± 0.212 ^Aa^	17.81 ± 0.95
	Location Mean ± SE	12.16 ± 0.412	18.65 ± 0.875	
pH	Afforested	7.59 ± 0.030 ^Aa^	8.05 ± 0.031 ^Ab^	7.82 ± 0.06
	Control	7.56 ± 0.043 ^Ba^	8.17 ± 0.027 ^Aa^	7.87 ± 0.08
	Location Mean ± SE	7.57 ± 0.026	8.11 ± 0.025	
EC (dS m^−1^)	Afforested	0.12 ± 0.007	0.18 ± 0.012	0.15± 0.01
	Control	0.13 ± 0.002	0.18 ± 0.005	0.16± 0.01
	Location Mean ± SE	0.13 ± 0.004 ^b^	0.18 ± 0.006 ^a^	
SOC (%)	Afforested	1.65 ± 0.014 ^Ba^	2.40 ± 0.254 ^Aa^	2.03 ± 0.15
	Control	0.61 ± 0.035 ^Ab^	0.79 ± 0.031 ^Ab^	0.70 ± 0.03
	Location Mean ± SE	1.13 ± 0.121	1.60 ± 0.223	
TN (%)	Afforested	0.10 ± 0.005 ^Ba^	0.19 ± 0.014 ^Aa^	0.15 ± 0.01
	Control	0.03 ± 0.002 ^Ab^	0.04 ± 0.001 ^Ab^	0.04 ± 0.00
	Location Mean ± SE	0.07 ± 0.009	0.12 ± 0.018	
C/N	Afforested	16.22 ± 0.756	12.61 ± 0.593	14.42 ± 0.63 ^b^
	Control	22.74 ± 0.985	18.72 ± 0.356	20.73 ± 0.69 ^a^
	Location Mean ± SE	19.48 ± 0.961 ^a^	15.67 ± 0.778 ^b^	
CaCO_3_ (%)	Afforested	4.62 ± 1.660 ^Aa^	26.66 ± 2.780 ^Ba^	15.64 ± 2.98
	Control	4.85 ± 0.608 ^Aa^	32.07 ± 1.920 ^Bb^	18.46 ± 3.27
	Location Mean ± SE	4.74 ± 0.859 ^b^	29.37 ± 1.760 ^a^	
C_mic_	Afforested	183 ± 2.950 ^Ab^	323 ± 20.500 ^Bb^	253 ± 19.00
	Control	35.1 ± 1.090 ^Aa^	59.0 ± 2.060 ^Aa^	47.1 ± 2.97
	Location Mean ± SE	109 ± 17.000	191 ± 31.900	
MR	Afforested	0.62 ± 0.049 ^Ba^	1.30 ± 0.075 ^Aa^	0.96 ± 0.090
	Control	0.25 ± 0.008 ^Aa^	0.35 ± 0.011 ^Ab^	0.30 ± 0.013
	Location Mean ± SE	0.43 ± 0.048	0.82 ± 0.115	
*q*Mic	Afforested	1.11 ± 0.023	1.39 ± 0.065	1.25 ± 0.047 ^a^
	Control	0.59 ± 0.026	0.76 ± 0.034	0.67 ± 0.028 ^b^
	Location Mean ± SE	0.85 ± 0.062 ^b^	1.07 ± 0.081 ^a^	
*q*CO_2_	Afforested	3.37 ± 0.270 ^Bb^	4.06 ± 0.205 ^Ab^	3.72 ± 0.183
	Control	7.14 ± 0.237 ^Aa^	5.99 ± 0.296 ^Ba^	6.56 ± 0.227
	App. Mean ± SE	5.26 ± 0.467	5.02 ± 0.282	

Abbreviations: EC = electrical conductivity (dSm^−1^); TN = total soil N (%); SOC = soil organic carbon (%); C_mic_ = carbon in the microbial biomass (μg g^−1^); MR= microbial basal respiration (μg CO_2_–C g^−1^ soil h^−1^); *qMic* = microbial quotient; *q*CO_2_ = metabolic quotient. Significant at *p* < 0.05; Comparisons of the bedrock at each location are shown in capital letters, and comparisons of location levels at each bedrock level are shown in lowercase letters.

**Table 3:** *F* and *p-*values of the ANOVA for the effects of parent material, location, and their interactions on soil physicochemical, microbial properties, and stoichiometric indices.

Soil Properties	Models	*F*-Value	*p*-Value
Sand (%)	Parent material	99.46	<0.000
	Location	1516.62	<0.000
	Parent material x Location	15.75	<0.000
Clay (%)	Parent material	214.64	<0.000
	Location	295.11	<0.000
	Parent material x Location	31.26	<0.000
Silt (%)	Parent material	86.84	<0.000
	Location	157.88	<0.000
	Parent material x Location	10.71	0.002
pH	Parent material	2.08	0.158
	Location	258.03	<0.000
	Parent material x Location	5.63	0.023
EC (dS m^−1^)	Parent material	45.98	<0.000
	Location	1.33	0.257
	Parent material x Location	0.42	0.523
SOC (%)	Parent material	105.48	<0.000
	Location	13.21	0.001
	Parent material x Location	4.88	0.034
TN (%)	Parent material	208.18	<0.000
	Location	42.13	<0.000
	Parent material x Location	20.76	<0.000
C/N	Parent material	78.86	<0.000
	Location	28.78	<0.000
	Parent material x Location	0.08	0.778
CaCO_3_ (%)	Parent material	2.20	0.147
	Location	167.32	<0.000
	Parent material x Location	1.84	0.183
C_mic_	Parent material	389.60	<0.000
	Location	61.68	<0.000
	Parent material x Location	30.94	<0.000
MR	Parent material	209.29	<0.000
	Location	73.73	<0.000
	Parent material x Location	41.71	<0.000
*q*Mic	Parent material	205.99	<0.000
	Location	30.49	<0.000
	Parent material x Location	2.07	0.159
*q*CO_2_	Parent material	125.22	<0.000
	Location	0.84	0.364
	Parent material x Location	13.06	0.001

**Table 4:** Some descriptive statistics and *t*-test results of some growth parameters of afforestation made on different bedrocks.

Growth Characteristics	Parent Material	n	Minimum	Maximum	Mean ± SE	*t-*Values
DBH (cm)	Andesite	53	4.00	13.00	8.64 ± 0.314 ^b^	*** 5.68
	Limestone	64	6.00	15.00	10.92 ± 0.256 ^a^	
TH (m)	Andesite	53	3.00	6.20	4.92 ± 0.107 ^b^	*** 12.13
	Limestone	64	4.70	7.70	6.61 ± 0.091 ^a^	

(*** *p* ≤ 0.001. a, b; Comparisons of the parent materials are shown in lower-case letters) DBH: Diameter at Breast High (cm), TH: Tree Height (m).

**Table 5:** Standardized canonical discriminant function coefficients of soil properties and plant growth characteristics.

	Function		
Soil Variables	1	2	3
TH	1.195	0.330	0.384
DBH	1.233	−0.133	−0.510
Sand	−0.613	0.900	−0.258
Clay	−0.906	0.000	−0.483
pH	0.262	0.103	0.779
EC	−1.798	−0.646	−1.695
SOC	17.72	2.887	17.09
TN	0.936	−0.066	−2.746
C/N	−0.512	−0.197	−1.344
CaCO_3_	−0.030	−0.258	0.436
C_mic_	−14.30	−2.517	−11.11
MR	−0.434	0.412	0.607
*q*Mic(C_mic_/C_org_)	7.158	0.590	5.012
*q*CO_2_	0.958	0.069	0.004

## Data Availability

The original contributions presented in the study are included in the article, further inquiries can be directed to the corresponding author.
